# A two-step hierarchical hypothesis set testing framework, with applications to gene expression data on ordered categories

**DOI:** 10.1186/1471-2105-15-108

**Published:** 2014-04-14

**Authors:** Yihan Li, Debashis Ghosh

**Affiliations:** 1 Department of Statistics, Pennsylvania State University, University Park, State College, Pennsylvania 16802, USA

**Keywords:** Multiple testing, Overall false discovery rate, Mixed-directional false discovery rate, Benjamini-Hochberg procedure, Microarray, Time course, Dose response

## Abstract

**Background:**

In complex large-scale experiments, in addition to simultaneously considering a large number of features, multiple hypotheses are often being tested for each feature. This leads to a problem of multi-dimensional multiple testing. For example, in gene expression studies over ordered categories (such as time-course or dose-response experiments), interest is often in testing differential expression across several categories for each gene. In this paper, we consider a framework for testing multiple sets of hypothesis, which can be applied to a wide range of problems.

**Results:**

We adopt the concept of the overall false discovery rate (OFDR) for controlling false discoveries on the hypothesis set level. Based on an existing procedure for identifying differentially expressed gene sets, we discuss a general two-step hierarchical hypothesis set testing procedure, which controls the overall false discovery rate under independence across hypothesis sets. In addition, we discuss the concept of the mixed-directional false discovery rate (mdFDR), and extend the general procedure to enable directional decisions for two-sided alternatives. We applied the framework to the case of microarray time-course/dose-response experiments, and proposed three procedures for testing differential expression and making multiple directional decisions for each gene. Simulation studies confirm the control of the OFDR and mdFDR by the proposed procedures under independence and positive correlations across genes. Simulation results also show that two of our new procedures achieve higher power than previous methods. Finally, the proposed methodology is applied to a microarray dose-response study, to identify 17 *β*-estradiol sensitive genes in breast cancer cells that are induced at low concentrations.

**Conclusions:**

The framework we discuss provides a platform for multiple testing procedures covering situations involving two (or potentially more) sources of multiplicity. The framework is easy to use and adaptable to various practical settings that frequently occur in large-scale experiments. Procedures generated from the framework are shown to maintain control of the OFDR and mdFDR, quantities that are especially relevant in the case of multiple hypothesis set testing. The procedures work well in both simulations and real datasets, and are shown to have better power than existing methods.

## Background

With the rapid development of high-throughput technologies, large-scale experiments nowadays frequently adopt more complex designs, further complicating the issue of multiple testing. In designs where a single hypothesis is tested for each feature (e.g. testing differential expression between two treatments for each gene), we need to consider the adjustment of multiplicity across a large number of features. As experiments become more complicated, it is often the case that multiple hypotheses need to be tested for each feature. This creates two dimensions of multiplicity - one dimension comes from the multiple features (e.g. genes), while another dimension comes from the multiple hypotheses associated with each feature. In dealing with these multi-dimensional multiple testing problems, it is crucial to understand the underlying structure and adjust for multiplicity accordingly.

As an example of such problems, we consider gene expression studies over ordered categories. In these experiments, researchers are often interested in genes that are possibly differentially expressed across a number of time points or dose levels. In this case, multiple tests of differential expression are conducted for each gene. An easy way of adjusting for multiplicity would be to pool all the tests from all the genes together, and simply apply multiple testing procedures such as the Benjamini and Hochberg procedure (B-H procedure) [1], controlling for the false discovery rate (FDR). However, this approach ignores the fact that the two dimensions of multiplicity are not equivalent. A suitable approach should take into account the fact that the key goal in these experiments is to identify important genes, and that the false discoveries should be controlled at the gene level.

In recent years, many proposals have addressed these new issues in multiplicity arising in microarray time-course or dose-response experiments. Sun and Wei [2] considered the problem of multiple testing for pattern identification in these types of studies. They considered this a “set-wise” multiple testing problem, where each gene corresponds to a “set”. Since they focused on pattern identification, their methods test for specific patterns across time for each gene, instead of making multiple individual inferences of differential expression between the time points. Guo, Sarkar and Peddada [3] looked into the problem of controlling false discoveries when making multiple directional decisions for each gene, also for time-course and dose-response experiments. They considered individual inferences for each time point and proposed controlling a quantity called the mixed directional false discovery rate (mdFDR). We will later show that the main procedure introduced by Guo et al. [3] falls under the general framework of this paper. On the other hand, building upon our framework, we are able to easily come up with procedures that are more powerful than that proposed by Guo et al. [3].

Our approach is to consider the multiple hypotheses for each gene as belonging to a hypothesis set associated with that gene. We aim at controlling false discoveries on the hypothesis set level, but we also enable inferences on the individual hypotheses. Inspired by a procedure in Heller et al. [4] for testing differentially expressed gene sets, we discuss a general two-step procedure to first identify significant hypothesis sets, and to then make further individual tests on each of the hypotheses within the significant sets. The procedure adjusts for multiplicity in both steps by adopting the Benjamini-Hochberg procedure in the first step and family-wise error rate controlling procedures in the second step. To measure false discoveries on the set level, we adopt the concept of the overall false discovery rate (OFDR), which was introduced by Benjamini and Heller [5] in the context of screening for partial conjunction hypothesis and further discussed in Heller et al. [4]. The OFDR is a straightforward extension of the FDR to hypothesis sets. The general two-step procedure we discuss is proved to control the OFDR under independence between the hypothesis sets, which follows from a similar result in the paper by Heller et al. [4]. In addition, we extend the general procedure to incorporate the cases of making directional decisions for two-sided alternatives, and discuss the control of the mixed-directional FDR in this case.

We applied this framework to gene expression data on ordered categories and developed three procedures for the problem of identifying genes that are differentially expressed between categories, as well as making directional decisions for each significant expression change. We conducted a simulation study to show that all three proposed procedures maintain control of the OFDR and mdFDR at the desired level under independence between genes, as well as positive correlations across genes. Simulation results show that two of our new procedures perform better in terms of power compared to the procedure in Guo et al. [3]. The proposed methodology is also applied to a microarray dose-response experiment by Coser et al. [6] which studied 17 *β*-estradiol (E2) sensitivity of genes in breast cancer cells. We identified genes that are induced at low concentrations of E2, and compared the results across the different procedures. Our results confirmed and complemented the original findings.

## Methods

### A framework for multiple hypothesis set testing and a general two-step procedure

We first present a general framework for multiple hypothesis set testing.

Suppose we want to test for *m* sets of hypotheses *H*(1),…,*H*(*m*) simultaneously. In each set of hypotheses *H*(*i*), there is a screening hypothesis, denoted by *H*_0_(*i*), that will be tested first. The rest of the hypotheses in the set *H*_1_(*i*),…,*H*_*n*(*i*)_(*i*), which we will refer to as the individual hypotheses, will be tested simultaneously if and only if *H*_0_(*i*) is rejected. In general, the number of individual hypotheses in each set need not be the same. Note that all the hypotheses referred to here are by default null hypotheses.

In this formulation of multiple testing of hypothesis sets, we wish to control the proportion of false rejections on the level of the hypothesis sets. So instead of the usual false discovery rate, we consider the overall false discovery rate (OFDR) [5], which is a similar concept but defined in terms of the hypothesis sets. It is defined as the expected proportion of falsely rejected hypothesis sets out of all the rejected hypothesis sets. We define a hypothesis set to be rejected if the screening hypothesis in the set is rejected; and we define a hypothesis set to be falsely rejected if at least one true null hypothesis in the set (including the screening hypothesis) were incorrectly rejected. The formal definition of OFDR is given below.

#### **Definition 1**

A hypothesis set *H*(*i*) (*i*=1,…,*m*) is said to be rejected if the screening hypothesis *H*_0_(*i*) is rejected. A hypothesis set *H*(*i*) is said to be falsely rejected if it is rejected and at least one hypothesis in the set *H*_0_(*i*),*H*_1_(*i*),…,*H*_*n*(*i*)_(*i*) is falsely rejected. The overall false discovery rate (OFDR) is defined as

$$\text{OFDR}=E\left(\frac{V}{R\vee 1}\right),$$

where *R*∨1=*max*(*R*,1), *R* is the total number of hypothesis sets rejected out of *m*, and *V* is the total number of hypothesis sets that are falsely rejected out of *m*.

Heller et al. [4] proposed a two-step procedure for testing differentially expressed gene sets that controls the OFDR of the gene sets. Here we formally discuss a general two-step hierarchical testing procedure for the multiple hypothesis set testing framework. The general procedure proceeds as follows. Let *p*_*j*_(*i*) be the unadjusted p-value for individually testing the *j*th hypothesis in the *i*th set, where *i*=1,…,*m* and *j*=0,1,…*n*(*i*).

Procedure 1:

•Apply the Benjamini-Hochberg procedure at level *α* to the *m* p-values corresponding to the screening hypotheses *p*_0_(1),…,*p*_0_(*m*). Let *R* be the number of rejected screening hypotheses.

•For each rejected hypothesis set *H*(*i*), test for the individual hypotheses *H*_1_(*i*),…,*H*_*n*(*i*)_(*i*) simultaneously, applying a p-value adjusting procedure on *p*_1_(*i*),…,*p*_*n*(*i*)_(*i*) such that the family-wise error rate (FWER) of these *n*(*i*) tests are controlled at level *Rα*/*m*.

Procedure 1 enables two levels of inferences. We are able to identify the significant hypothesis sets by testing the screening hypotheses for each set. At the same time, for each significant hypothesis set, we are able to identify the significant individual hypotheses within the set. On the other hand, the false discovery rate is administered only on the set level. Procedure 1 controls the OFDR of testing the *m* hypothesis sets at level *α*, under the condition that the p-values of the individual hypotheses in each hypothesis set are independent from all other screening hypothesis p-values. The proof follows directly from the proof in [4] of a similar claim on their procedure. We state the theorem formally below.

#### **Theorem 1**.

Procedure 1 controls the overall false discovery rate (OFDR) at level *α* assuming that for each hypothesis set *H*(*i*), the p-values *p*_0_(*i*),*p*_1_(*i*),…,*p*_*n*(*i*)_(*i*) are independent of all the other screening p-values, *p*_0_(1),…,*p*_0_(*m*) excluding *p*_0_(*i*).

Though Theorem 1 only states the case of independence between the hypothesis sets, in practice, the framework can be applied to more general cases where certain positive dependency structures exist between the test statistics from different hypothesis sets. Intuitively, this is because Procedure 1 relies on the B-H procedure. The conditions on dependence such that the FDR is controlled are given in [7]. On the other hand, we will later present simulation studies that cover the situation where positive correlation exists across the hypothesis sets, and the results demonstrate control of OFDR in these cases

The multiple hypothesis set testing framework and the general two-step procedure have very broad applicability. They provide a general platform for dealing with practical problems in large scale experiments where simultaneous inference is needed on two or more dimensions. Take microarray experiments as a general example. One dimension of multiplicity would be the genes, while another dimension would represent the multiple inferences for each gene that the researcher is interested in. We now give some examples: 1) in time-course experiments, the second dimension could reflect hypotheses on/between the different time points; in dose-response experiments, the second dimension could reflect hypotheses on/between the different dose levels; 2) in experiments with multiple treatment groups, the second dimension could reflect a number of possible pairwise comparisons between treatments. In all of these cases, the multiple hypotheses for each gene would make up the hypothesis set for that gene. Thus each gene would correspond to a hypothesis set. Under this context, intuitively, we would want to control the false discoveries on the gene level, despite the multiple inferences made for each gene. This makes the OFDR a reasonable quantity to control, compared to the usual FDR. In general, our framework and procedure can be reasonably adapted to any multi-dimensional multiple testing problem that has a hierarchical structure where it makes sense to define hypothesis sets.

### Incorporating directional decisions

In many practical settings, following the rejection of a hypothesis, researchers are interested in making additional claims. In the case of two-sided alternatives, directional decisions are often made. This is especially common in differential expression analysis of genomic data, where it is important to claim the direction of expression change after finding a difference. In these situations, in addition to the traditional type I error, there is also a chance of making directional errors [3,8]. Directional errors occur when a two-sided test is correctly rejected, but the choice of the alternative (directional decision) is incorrect. If directional decisions are desired, it is important to take into account directional errors (sometimes referred to as type III errors [8]) in additional to type I errors.

Guo et al. [3] discussed the idea of mixed-directional FDR (mdFDR), which is similar in concept to the OFDR, but with the addition of directional errors. We give the formal definition of mdFDR below.

#### **Definition 2**.

The mixed-directional false discovery rate (mdFDR) is defined as

$$\text{mdFDR}=E\left(\frac{V+S}{R\vee 1}\right),$$

where *R*∨1= max(*R*,1), *R* is the total number of hypothesis sets rejected out of *m*, *V* is the total number of hypothesis sets that are falsely rejected out of *m*, and *S* is the total number of hypothesis sets that are correctly rejected but for which at least one directional error was made when making directional decisions for the individual hypotheses.

It is apparent that OFDR≤mdFDR. Thus while the general Procedure 1 guarantees the control of the OFDR under independence conditions (by Theorem 1), it does not automatically guarantee the control of the mdFDR. However, we can easily extend the proof of Theorem 1 to obtain the following results.

#### **Lemma 1**.

Under the assumptions of Theorem 1, Procedure 1 controls the mixed-directional FDR (mdFDR) at level *α* if the family-wise error rate controlling procedure used in step (2) maintains control of both type-I and directional errors.

Although Lemma 1 is just a direct extension of Theorem 1, it provides great practical benefits. This is because many commonly used family-wise error rate controlling procedures have been shown to maintain control of directional errors under certain conditions [8]. For instance, Finner [8] showed that the Bonferroni procedure, Holm’s procedure, as well as Hochberg’s procedure are all able to control both type I and directional errors family-wise, for multiple two-sided tests involving independent T-statistics. This covers the most common two-sided tests as well as some of the most common family-wise error rate controlling procedures. Thus Lemma 1 allows us to extend Procedure 1 to many situations where directional decisions are needed, and ensures control of the mdFDR in addition to the OFDR

### Procedures for testing gene expression differences on ordered categories

Now we shall introduce methods for testing differential expression for microarray experiments on ordered categories, while controlling for the OFDR/mdFDR on the gene level. The procedures we are proposing are based on the hypothesis set testing framework and the general Procedure 1 described previously.

In microarray experiments on ordered categories, such as time-course or dose-response experiments, depending on the study design, some of the common research interests include discovering: (i) genes that are differentially expressed between two treatment groups at certain time points or dose levels; (ii) genes that are differentially expressed between successive time points or dose levels; or (iii) genes that are differentially expressed at certain time points or dose levels compared to a starting time or baseline dose level. Regardless of the specific case, the commonality is that we are interested in testing multiple hypotheses simultaneously for each gene, and that a gene would be considered interesting if at least one of its associated hypotheses is significant.

The cases described above are problems of multiple hypothesis set testing, where each gene corresponds to a hypothesis set. The individual hypotheses of each hypothesis set are the multiple hypotheses that are tested for each gene. On the other hand, the screening hypothesis for each hypothesis set would test an overall hypothesis of whether the gene is differentially expressed at all. For the case of time-course and dose-response experiments, it would be reasonable to set up the screening hypothesis such that it tests for the conjunction of the individual hypotheses. That is, for each hypothesis set, the screening hypothesis tests for whether at least one of the individual null hypotheses can be rejected.

Assume that we test the same set of *q* individual hypotheses for each gene, i.e. *n*_(*i*)_=*q* for *i*=1,…,*m*. Let *H*_1_(*i*),…,*H*_*q*_(*i*) denote the individual null hypotheses for gene *i*, *i*=1,…,*m*. The screening null hypotheses for gene *i* is $H_{0}(i)=\cap_{k=1}^{q}H_{k}(i)$, which is the conjunction of the individual hypotheses. Let *p*_1_(*i*),…,*p*_*q*_(*i*) denote the p-values for testing *H*_1_(*i*),…,*H*_*q*_(*i*) individually. There are many possible methods for testing the conjunction of hypotheses in order to obtain *p*_0_(*i*) for the screening hypothesis *H*_0_(*i*), including more conservative family-wise error rate controlling methods such as the Bonferroni method, or commonly used meta-analysis methods such as Fisher’s combined probability test. In this case though, referring to Procedure 1, since we ultimately need to make inference on each of the individual hypothesis in the second step, it makes sense to use methods for testing *H*_0_(*i*) such that the rejection of *H*_0_(*i*) leads to at least one rejection out of *H*_1_(*i*),…,*H*_*q*_(*i*). Thus meta-analysis methods such as the Fisher’s combined probability test that do not have corresponding procedures for making inference on the individual hypotheses are not suitable in this case. On the other hand, multiple testing procedures such as the Bonferroni method, Holm’s step-down procedure [9] or Hochberg’s step-up procedure [10], can be used to test for the conjunction of hypotheses and at the same time test for the individual hypotheses while controlling the family-wise error rate. Based on Procedure 1 and the three family-wise error rate controlling methods mentioned, we propose the following three procedures for making inference on gene expression data on ordered categories. Let *p*_(1)_(*i*)≤⋯≤*p*_(*q*)_(*i*) be the ordered versions of *p*_*j*_(*i*), *j*=1,…,*q*, for a fixed *i*.

Procedure 2:

•Based on Bonferroni’s method, let the screening p-value *p*_0_(*i*)=*qp*_(1)_(*i*) for *i*=1,…,*m*. Apply the Benjamini-Hochberg procedure at level *α* to *p*_0_(1),…,*p*_0_(*m*) for testing *H*_0_(1),…,*H*_0_(*m*) simultaneously. Let *R* be the number of rejected screening hypotheses.

•For every *j*=1,…,*q* and *i*=1,…,*m* with *p*_*j*_(*i*)≤*Rα*/(*qm*), reject the corresponding *H*_*j*_(*i*). If desired, the directions of expression changes can be declared according to the signs of the test statistics.

Procedure 3:

•Based on Holm’s method, let the screening p-value *p*_0_(*i*)=*qp*_(1)_(*i*) for *i*=1,…,*m*. Apply the Benjamini-Hochberg procedure at level *α* to *p*_0_(1),…,*p*_0_(*m*) for testing *H*_0_(1),…,*H*_0_(*m*) simultaneously. Let *R* be the number of rejected screening hypotheses.

•For every *i*=1,…,*m*, let *R*_*i*_= max{1≤*j*≤*q*: *p*_(*l*)_(*i*)≤*Rα*{*m*(*q*+1−*l*)}^−1^, for *l*=1,…,*j*}, if the maximum exists; otherwise *R*_*i*_=0. For every *i* and *j* with *p*_*j*_(*i*)≤*Rα*{*m*(*q*+1−*R*_*i*_)}^−1^ (or equivalently $p_{j}(i)\leq p_{\left(R_{i}\right)}(i)$), reject the corresponding *H*_*j*_(*i*). If desired, the directions of the expression changes can be declared according to the signs of the test statistics.

Procedure 4:

•Based on Hochberg’s method, let the screening p-value *p*_0_(*i*)= min1≤*j*≤*q*{(*q*+1−*j*)*p*_(*j*)_(*i*)} for *i*=1,…,*m*. Apply the Benjamini-Hochberg procedure at level *α* to *p*_0_(1),…,*p*_0_(*m*) for testing *H*_0_(1),…,*H*_0_(*m*) simultaneously. Let *R* be the number of rejected screening hypotheses.

•For every *i*=1,…,*m*, let *R*_*i*_= max{1≤*j*≤*q*: *p*_(*j*)_(*i*)≤*Rα*{*m*(*q*+1−*j*)}^−1^}, if the maximum exists; otherwise *R*_*i*_=0. For every *i* and *j* with *p*_*j*_(*i*)≤*Rα*{*m*(*q*+1−*R*_*i*_)}^−1^ (or equivalently $p_{j}(i)\leq p_{\left(R_{i}\right)}(i)$), reject the corresponding *H*_*j*_(*i*). If desired, the directions of expression changes can be declared according to the signs of the test statistics.

By Theorem 1, Procedures 2, 3 and 4 control the OFDR of the genes under independence between genes and other conditions required for Bonferroni’s, Holm’s and Hochberg’s methods to control the family-wise error rate. In addition, by Lemma 1, Procedures 2, 3 and 4 also maintain control of the mdFDR of the genes under independence between the genes and the test statistics for the individual hypotheses for each gene, as well as the conditions required for Bonferroni’s, Holm’s and Hochberg’s methods to control the family-wise error rate. In fact, Bonferroni’s method and Holm’s method control the family-wise error rate without any restrictions on the individual hypotheses. Hochberg’s method does require either independence between the individual hypotheses or certain positive dependence structures (more details on this can be found in [11]). On the other hand, since Hochberg’s method is uniformly more powerful than Holm’s method, which is uniformly more powerful than Bonferroni’s method, Procedure 4 is more powerful than Procedure 3, which in turn is more powerful than Procedure 2. Notice though that the screening p-values for Procedure 2 and 3 are the same, so that they would reject the same genes in step one, but Procedure 3 would potentially find more significant individual hypotheses in step two compared to Procedure 2.

Interestingly, the main procedure proposed by Guo et al. [3] for making multi-dimensional directional decisions is essentially the same as Procedure 2 above. In an attempt to increase power, Guo et al. [3] also proposed another procedure similar in structure but using the Simes method [12] instead of the Bonferroni method. However, as discussed in [3], the procedure based on the Simes method, though potentially more powerful, does not guarantee control of the mdFDR. Under our proposed framework, it is easy to see that the problem lies within the fact that the Simes method does not guarantee control of the family-wise error rate, which is a required property of the method used in the second step, as seen in the general Procedure 1. With this observation in mind, the key to improving power over the procedure in Guo et al. [3], is to use family-wise error rate controlling methods that are more powerful than Bonferroni’s method, naturally leading to Procedures 3 and 4.

## Results and discussion

### A simulation study

We conducted a simulation study to illustrate the control of the OFDR and mdFDR of Procedures 2, 3 and 4, as well as compare their performances on power. We shall set up the simulation study exactly following the paper by Guo et al. [3]. Since Procedure 2 is essentially the same as the procedure proposed in [3], this allows us to directly compare the performances of our new Procedures 3 and 4 with their procedure.

Following [3], we simulate the setting of a time-course experiment with *m*=1000 genes, and 6 time points. We are interested in differential expression between successive time points, which leads to *q*=5 hypotheses for each gene. The gene expression vectors ***Z***_*j*_ (*j*=1,…,6) for the 6 time points are simulated from independent *m*-dimensional multivariate normal distributions, where *Z*_*j* *i*_∼*N*(*μ*_*j* *i*_,1) (*i*=1,…,*m*) and have a common correlation *ρ*. *ρ* is set to be 0, 0.2, 0.5 or 0.8 for four separate simulations respectively. Let the vector of expression differences between successive time points for each gene *i* be ***δ***_*i*_, where each component $\delta_{\text{ij}}=(\mu_{j+1,i}-\mu_{\text{ji}})/\sqrt{2}$ for *j*=1,…,5. Out of the *m* ***δ***_*i*_’s, *m*_0_ were set to a zero vector, and the *δ*_*i* *j*_’s in 50*%*, 25*%* and 25*%* of the remaining *m*−*m*_0_ ***δ***_*i*_’s were randomly generated (uniformly) from the intervals (−0.75,0.75), (−4.25,−2.75) and (2.75,4.25) respectively. The null hypothesis tested is *δ*_*i* *j*_=0 for all *i* and *j*. The test statistic for testing each *δ*_*i* *j*_ is $T_{\text{ij}}=(Z_{j+1,i}-Z_{\text{ji}})/\sqrt{2}$ and the corresponding p-value is computed by *p*_*i* *j*_=2{1−*Φ*(|*Tij*|)}, where *Φ*(·) is the standard normal CDF. Here *p*_*i* *j*_ are the p-values for the individual hypotheses for each gene *i* - corresponding to the notation of *p*_*j*_(*i*) used in the methods section. This simulation set up, as well as the parameter values, strictly follow that of [3]. Simulation results are averaged across 1000 replications. The level *α* is set to be 0.05. Notice that even though theory on all the procedures were developed under independence between genes, we also investigate the cases where genes are positively correlated in the simulation study.

We consider Procedures 2, 3 and 4 in our simulation study. As a comparison to these two-step procedures, we also consider what we call the simple B-H procedure, which is basically a one-step procedure that simply tests for the *mq* individual hypotheses simultaneously by directly applying the Benjamini-Hochberg procedure. By construction, the simple B-H method would control the FDR of the *mq* individual hypotheses, but it would be interesting to see how it performs with respect to the gene-wise OFDR or mdFDR. The simple B-H method does not conduct tests on the hypothesis set level, but in order to compare it with the two-step procedures, we can define a hypothesis set (or gene) to be rejected if any of its individual hypotheses are rejected by the simple B-H method, and define the OFDR/mdFDR correspondingly.

**Figure 1:**
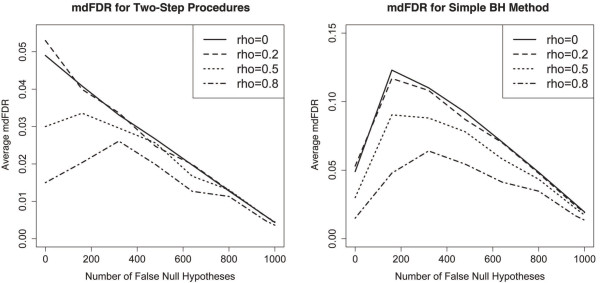
**Evaluation of the mixed directional FDR.** Evaluation of the mixed directional FDR (*α*=0.05). Each plot includes results for the four different correlations settings (among genes): *ρ*=0, 0.2, 0.5 and 0.8. The first plot shows the control of mdFDR for Procedures 2, 3 and 4 (only one set of results are reflected because the mdFDR for the three procedures are almost exactly identical). The second plot shows the non-control of mdFDR for the simple B-H method.

In this simulation, evaluating the mdFDR would be more appropriate, since we do care about the direction of change across the time points. Figure 1 shows the evaluation of the mdFDR for the different methods and correlation settings. Results for the OFDR are very similar to those of the mdFDR in our simulations. We do not provide separate plots for the OFDR, but note that since OFDR≤mdFDR, the OFDR is controlled whenever the mdFDR is controlled. The first plot in Figure 1 shows the control of the mdFDR (for *α*=0.05) by Procedures 2, 3 and 4. These three procedures have almost exactly identical results for mdFDR, thus only one set of results are reflected in the plot. Notice that the mdFDR is not only controlled under independence between genes (*ρ*=0), which is proved by theory, but it is also controlled under the three positive correlation settings (*ρ*=0.2,0.5,0.8). In fact, it seems that stronger positive correlation results in even lower mdFDR. The plot also shows that the mdFDR decreases as the number of false null hypotheses increases. More precisely, the x-axis is the number of genes (or hypothesis sets) for which at least one null hypotheses is false. For these genes, which we shall call “false null genes”, the screening null hypothesis associated with it would be false. Notice that as the number of false null genes reaches 1000 (i.e., all the genes are false null genes), the mdFDR does not decrease to 0. In this case, even though there would be no probability of making false discoveries with regard to the screening hypotheses (since all screening hypotheses are false), there is still a positive probability of making false discoveries, especially false directional decisions for the individual hypotheses. The second plot in Figure 1 shows the average mdFDR for the simple B-H method. As we can see, the simple B-H method fails to control the mdFDR at *α*=0.05. This illustrates the fact that the FDR with respect to the *m*×*q* individual hypotheses and the OFDR/mdFDR with respect to the *m* hypothesis sets are distinct concepts. It can be shown that the simple B-H method always rejects at least as many genes as Procedure 2. However, the rejections by simple B-H method are on the basis of the individual hypotheses - not considering each gene as an entity. In this case, if the interest is in controlling the false discoveries of the genes, then simply applying an FDR controlling procedure to all the tests does not guarantee the control of the desired OFDR/mdFDR.

Next, we shall look at the performances on power for the different procedures. We first need to define power in the context of multiple hypothesis set testing. In general multiple testing problems, we evaluate power by looking at the proportion of false null hypotheses that are correctly rejected by a method. We can adopt this definition of power for our problem as well, if we put aside the hypothesis sets for a moment, and directly look at all the *m*×*q* individual hypotheses. We shall name this “power (I)”. On the other hand, we can define power with respect to the hypothesis sets, by looking at the proportion of false null genes that are correctly rejected. Here, false null genes refer to the genes for which at least one null hypotheses is false, as mentioned previously. Further, we say that a false null gene is correctly rejected, if and only if a correct decision is made for every single null hypothesis for that gene - i.e., we need to correctly reject every false null hypothesis for that gene, and at the same time not reject any true null hypothesis for that gene. The power defined this way is with respect to the hypothesis sets and we shall name it “power (II)”.

**Figure 2:**
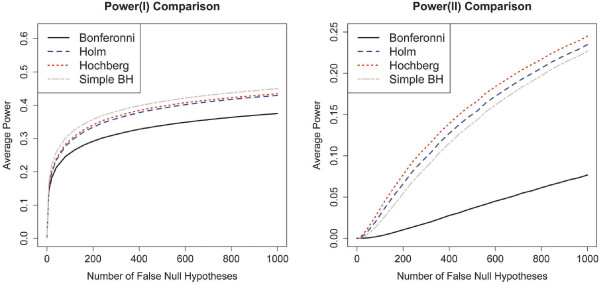
**Comparison of power.** Comparison of power for Procedures 2, 3 and 4, and the simple B-H method. Power (I) is with respect to the *m*×*q* individual hypotheses. Power (II) is with respect to the *m* hypothesis sets (genes). In the legend, “Bonferroni” refers to Procedure 2, the same as that proposed in Guo et al. (2010). “Holm” and “Hochberg” refer to Procedures 3 and 4 respectively, which are our newly proposed procedures. Results here are for *ρ*=0. The other correlations settings have very similar results.

Figure 2 shows the simulation results for power (I) and (II) respectively for *ρ*=0. Results for other cases of *ρ* are not shown here, but they look very similar to the case of *ρ*=0. For both definitions of power, the results are compared across four methods: Procedure 2 (based on Bonferroni), Procedure 3 (based on Holm), Procedure 4 (based on Hochberg), and the simple B-H method. As mentioned before, Procedure 2 (Bonferroni) is the same as the procedure proposed in [3], while Procedures 3 (Holm) and 4 (Hochberg) are newly proposed procedures. We can see that Procedures 3 (Holm) and 4 (Hochberg) show considerable improvements in power compared to Procedure 2 (Bonferroni), especially for power (II), which is defined with respect to the hypothesis sets. This result is to be expected, as mentioned earlier, since Holm’s and Hochberg’s method are more powerful than Bonferroni’s method. It is worth noting that even though Procedure 3 (Holm) selects the same genes in the first step as Procedure 2 (Bonferroni), it still results in a much higher power (II) (which is defined with respect to the genes). This is because Procedure 3 correctly rejects more false individual null hypotheses. We also see that Procedure 4 (Hochberg) has a slight gain in power over Procedure 3 (Holm), but this comes at the price of restrictions on the dependence structure among the individual hypotheses. On the other hand, the performance of the simple B-H method is interesting. In general, the power of the simple B-H method is similar to that of Procedure 3 (Holm) and 4 (Hochberg). For power (I), the simple B-H method performs the best. This is not very surprising, since power (I) is the power with respect to the *m*×*q* individual hypotheses. But the simple B-H method does not perform as well for power (II), which is defined with respect to the hypothesis sets. This again reinforces the idea that it is very different as to whether we treat the problem simply as one of multiple testing of *mq* individual hypotheses, or look at it from the gene-wise point of view and treat it as multiple hypothesis set testing of *m* hypothesis sets.

In summary, results from the simulation study show that Procedures 2, 3 and 4 do indeed control the OFDR and mdFDR under independence between genes, as well as positive correlation between genes. The three procedures perform almost identically with regards to the mdFDR/OFDR. On the other hand, our new Procedures 3 and 4 show considerable gains in power compared to Procedure 2 (as in [3]). By comparing the two-step procedures with the simple B-H method, we also gain some insight into the differences between treating a problem as a simple multiple testing problem versus a multiple hypothesis set testing problem.

### An application to a microarray dose-response experiment

Coser et al. [6] studied the effect of estrogen on gene expression in breast cancer cells. In particular, they are interested in characterizing the effect of low concentrations of 17 *β*-estradiol (E2) on the transcriptome profile of MCF7/BUS human breast cancer cells. According to [6], the E2 dose-dependent growth curve of these cells saturate with 100 pM E2, which is a concentration unlikely to be maintained *in vivo*. Thus it is important to study the effects of lower, unsaturated concentrations of E2. Varying low concentrations of E2 are investigated in this study through a microarray dose-response experiment using Affymetrix U-133A chips. Gene expressions are evaluated at 5 different levels of concentration of E2: 0, 10, 30, 60 and 100 pM. Five replicates are used for each concentration. Gene expressions are evaluated for a total of 22283 genes. The gene expression dataset from this study can be found in the NCBI GEO public database under the record number GDS2324.

We apply the methodology we proposed in this article to the dose-response gene expression data from [6] to identify genes that are significantly induced or suppressed at various low concentrations of E2. More specifically, we test four individual hypotheses for each gene, to detect differential expression at 10, 30, 60 and 100 pM compared to 0 pM respectively. The screening hypothesis for each gene tests for whether the gene is differentially expressed at all at any of the four levels of concentration of E2 compared to absence of E2. The ability of our procedure to make directional decisions is utilized to decide whether the significantly differentially expressed genes are induced or suppressed.

We tried all three procedures on the data: Procedure 2 (Bonferroni), which corresponds to that of [3], and Procedures 3 (Holm) and 4 (Hochberg). Theoretically, in order to control the OFDR, Procedure 4 (Hochberg) requires that the tests between the multiple dose levels and 0 pM be independent or satisfy certain positive dependence criteria. It seems reasonable to assume that these tests within each gene are positively correlated, therefore we think it is appropriate to apply Procedure 4 here in practice.

With the overall false discovery rate controlled at level 0.05, Procedures 2 and 3 identified 368 genes that are differentially expressed at some level of E2 (of which 204 were induced and 164 were suppressed), while Procedure 4 identified 374 genes (of which 208 were induced and 166 were suppressed). Note that in this application, the significant individual hypotheses within a gene always display consistent direction of change, regardless of which procedure was used, thus enabling us to declare each significant gene as induced or suppressed. Recall that Procedures 2 and 3 will always reject the same hypothesis sets, but may not reject the same individual hypotheses subsequently. With regards to the individual hypotheses, the number of individual hypotheses deemed significant by Procedures 2, 3 and 4 are 579, 640 and 662 respectively. Notice that our new procedures were able to detect a considerably larger number of significant individual hypotheses.

The implications of the fact that Procedures 2 and 3 reject the same genes but that Procedure 3 rejects more individual hypotheses is that, for the same genes identified by both procedures, Procedure 3 is more likely to detect significant differential expression for a lower concentration level of E2, thus better characterizing the sensitivity of the genes. Table 1 summarizes the distribution of the number of E2 concentration levels that the identified genes are found to be differentially expressed in, for the three procedures respectively. As we can see, out of the 368 genes identified by both Procedures 2 and 3, only one gene was found to be differentially expressed at all four concentrations of E2 by Procedure 2, while nine of them were identified by Procedure 3 to be differentially expressed at all four concentration levels. On the other hand, 200 genes were found to be differentially expressed at only one concentration of E2, according to Procedure 2. However, 28 of them were found to be also differentially expressed at other concentration levels by Procedure 3. To further illustrate this point, we compare the genes that are identified by the three procedures as having very high E2 sensitivity (induced or suppressed at 10 pM E2). Procedures 3 and 4 detected 9 and 10 genes respectively that are induced at 10 pM E2, while Procedure 2 only detected 3 of them. In particular, progesterone receptor gene PGR, one of the genes found to have very high sensitivity by [6], was identified by Procedures 3 and 4, but not by Procedure 2. At the same time, Procedures 3 and 4 detected 6 and 8 genes suppressed at 10 pM E2, while Procedure 2 detected only 4. Again, one of the genes found to be E2-suppressible by [6], apolipoprotein D gene APOD, was only detected by Procedures 3 and 4 but not by Procedure 2. These results show that our new procedures are better options for this problem compared to that of [3].

**Table 1:** Distribution of the number of dose levels that the identified genes are found to be differentially expressed in

**Method**	**1**	**2**	**3**	**4**	**Total**
Proc. 2 (Bonferroni)	200	126	41	1	368
Proc. 3 (Holm)	172	129	58	9	368
Proc. 4 (Hochberg)	171	130	61	12	374

The direct comparison of the lists of genes found by our procedures and those in [6] is not straightforward, partly due to the fact that [6] considered both the p-values and the fold-changes in determining significance, but without making any explicit adjustments to multiple testing. However, to compare the pathways associated with the gene lists, we performed functional annotation clustering analysis using DAVID, which is available at http://david.abcc.ncifcrf.gov/home.jsp. The analysis was performed on lists of genes with high E2 sensitivity that were induced at concentrations 10 pM or 30 pM. We compare the results from Procedures 2 and 3. We did not include Procedure 4 because its gene list is rather similar to that of Procedure 3. The top five groups of functions associated with each genes lists are summarized in Table 2 along with corresponding enrichment scores. The top functions identified by the three gene lists are fairly similar, showing a consistency in findings, especially compared to that of [6]. On the other hand, the enrichment scores associated with the results produced by Procedure 3 are the highest among the three. This indicates that the evidence gathered by Procedure 3 for the top functions is stronger.

**Table 2:** Summary of top functions and corresponding enrichment scores from functional annotation clustering results by DAVID for gene lists (inducible genes at low concentrations of E2) from three different methods

**Enrichment score**	**Top functions by Coser et al. (2003)**
4.11	Cell cycle, cell division, intracellular non-membrane-bounded organelle
2.48	DNA metabolic process, DNA repair, DNA recombination, cellular response to stress, disease mutation,
	response to DNA damage stimulus/ionizing radiation/abiotic stimulus
2.42	Chromosome organization, M phase of meiotic cell cycle
2.31	Microtubule-based process, centrosome cycle, microtubule cytoskeleton, enzyme binding
1.9	DNA replication, regulation of cell cycle, microtuble cytoskeleton, nuclear lumen, negative regulation
	of nucleobase/nitrogen compound/macromolecule metabolic process
	**by Procedure 2 (Bonferroni)**
4.56	DNA replication, DNA metabolic process, nucleoplasm
2.95	Response to DNA damage stimulus, cellular response to stress, DNA repair
2.72	Nucleoplasm, nuclear lumen, intracellular organelle lumen
2.16	Cholesterol biosynthesis and metabolic process, lipid synthesis and metabolic process, sterol biosynthesis
	and metabolic process, isoprenoid biosynthetic/metabolic process
2.01	Chromosome, intracellular non-membrane-bounded organelle
	**by Procedure 3 (Holm)**
5.85	Chromosome, intracellular non-membrane-bounded organelle
4.85	DNA replication, DNA metabolic process, DNA-dependent ATPase MCM, nucleoplasm, intracellular organelle
	lumen, purine nucleotide binding, adenyl robonucleotide binding
3.66	Response to DNA damage stimulus, DNA repair, cellular response to stress
3.13	Chromosome part, nuclear chromosome part
3.1	Cell cycle, cell division, mitosis, condensed chromosome, M phase, kinetochore, organelle fission

## Conclusions

In large-scale experiments, such as microarray gene expression studies, as the problem and the designs become more complicated, new issues in multiple testing arise. For instance, in microarray time-course or dose-response experiments, in addition to considering tens of thousands of genes simultaneously, multiple hypotheses are often being tested for each gene. As a result, the problem of multiplicity becomes multi-dimensional. Traditional concepts of type I error control and methods for large-scale multiple testing (e.g. the FDR and the Benjamini-Hochberg procedure) can still be used, but may not be optimal for these more complex designs. Hence, it is important to consider new measures of type I error and develop statistical methods for these multi-dimensional multiple testing problems.

The methodology in this article provides one way of approaching these problems. We have formulated certain types of multi-dimensional multiple testing problems as multiple hypothesis set testing. In the case of microarray time-course/dose-response experiments, we consider each gene to be associated with a hypothesis set, where the multiple individual hypotheses in the set test for differential expression among a number of different time points or dose levels. We have adopted the concept of the overall FDR, which is a measure of the FDR on the hypothesis set level. By doing so, we aim at controlling the false discoveries on the gene level, which increases the interpretability of the results, compared to focusing on the FDR of all the individual hypotheses. We discussed a general two-step hierarchical testing procedure for multiple hypothesis set testing, which is proved to control the OFDR under independence across the hypothesis sets. We also extended the general procedure to enable directional decisions for two-sided tests and discussed the control of the mdFDR under certain conditions. We then suggested three specific procedures for microarray time-course/dose-response experiments. These procedures not only allow us to test for differential expression across multiple time points or dose levels, but are also capable of identifying the direction of expression change, while still maintaining control of the OFDR and mdFDR. We evaluated the performance of the proposed procedures under both independence and dependence between genes and compared the power with previous methods. Finally, the methodology is applied to analyze data from a microarray dose-response study to identify genes that are differentially expressed at low concentrations of estrogen in breast cancer cells.

The key point in the hypothesis set testing framework is that the two-dimensional multiplicity is transformed into a hierarchical structure. Hypotheses are tested in the unit of sets in the first step. This is realized by the formulation of a screening hypothesis for each set. The first step of our procedures deals with the hypothesis sets much like dealing with a traditional multiple testing problem. By applying the Benjamini-Hochberg procedure to the screening hypotheses, we are able to adjust for part of the multiplicity on the hypothesis set level. Additional type I errors (and sometimes directional errors) that can potentially occur while making inference for the individual hypotheses in each set are controlled in the second step by applying family-wise error rate controlling procedures. Together, the OFDR (or mdFDR) is controlled at the hypothesis set level.

Although our focus was on applications to gene expression data over ordered categories, the proposed methodology is widely applicable. The framework of multiple hypothesis set testing is very flexible and can be easily adapted to many large-scale multiple testing problems with complex designs. For example, the methodology can be applied to microarray studies with ANOVA designs that require follow-up pairwise comparisons. In this case, each gene would still be associated with a hypothesis set, where the individual hypotheses in the set are the multiple pairwise comparisons between the number of treatments. On the other hand, it would be interesting to develop more powerful procedures for each specific type of problem. For example, if a large proportion of individual hypotheses are expected to be significant given the significance of the hypothesis set, then we can potentially improve power by incorporating adaptive multiple testing methods into the procedure. Much future work can be done on adapting the hierarchical hypothesis set testing framework and procedures to different multi-dimensional multiple testing problems.

## Competing interests

The authors declare that they have no competing interests.

## Authors’ contributions

YL developed the procedures, carried out the simulation studies and data analysis, and drafted the manuscript. DG supervised and contributed important ideas throughout the research process and revised the manuscript. Both authors read and approved the final manuscript.
